# Supplementary material for: Factors associated with underweight, overweight, and obesity in reproductive age Tanzanian women
Source: PLoS One. 2020 Aug 24;15(8):e0237720. doi: 10.1371/journal.pone.0237720 (PMC7444815; doi:10.1371/journal.pone.0237720)
Supplement: S1 Table — (DOCX) [file pone.0237720.s001.docx]

**S1 Table. Characteristic of the study participants of reproductive age Tanzanian women, TDHS 2015–16**

| **Variables** | **Frequency*** | **Percentage** |
| --- | --- | --- |
| **Socioeconomic factors** |  |  |
| Women’s education |  |  |
| No schooling | 1668 | 14.2 |
| Primary education | 7229 | 61.6 |
| Secondary education and higher | 2841 | 24.2 |
| Women’s employment |  |  |
| No employment | 2712 | 43.7 |
| Formal employment | 941 | 15.2 |
| Informal employment | 2556 | 41.2 |
| Marital status |  |  |
| Not married | 3188 | 27.2 |
| Currently married | 6966 | 59.3 |
| Formerly married | 1585 | 13.5 |
| Household wealth status |  |  |
| Poor | 3838 | 32.7 |
| Middle | 2078 | 17.7 |
| Rich | 5822 | 49.6 |
| **Demographic factors** | | |
| Women’s age |  |  |
| 15–24 years | 4689 | 40.0 |
| 25–34 years | 3312 | 28.2 |
| 35–49 years | 3737 | 31.8 |
| Parity |  |  |
| None | 3055 | 26.0 |
| 1–4 children | 5856 | 49.9 |
| 5+ children | 2826 | 24.1 |
| **Behavioural factors** | | |
| Listening radio |  |  |
| No | 2540 | 21.6 |
| Yes | 9198 | 78.4 |
| Read magazine |  |  |
| No | 6518 | 55.5 |
| Yes | 5220 | 44.5 |
| Watch television |  |  |
| No | 5454 | 46.5 |
| Yes | 6285 | 53.5 |
| Alcohol use |  |  |
| No | 9826 | 83.7 |
| Yes | 1913 | 16.3 |
| Smoking |  |  |
| No | 10686 | 99.6 |
| Yes | 49 | 0.4 |
| Contraceptive use |  |  |
| No | 7469 | 63.6 |
| Yes | 4269 | 36.4 |
| **Community-level factors** | | |
| Place of residence |  |  |
| Urban | 4330 | 36.9 |
| Rural | 7408 | 63.1 |
| Region of residence |  |  |
| Western zone | 1082 | 9.2 |
| Northern zone | 1431 | 12.2 |
| Southern highlands | 735 | 6.3 |
| Southern zone | 644 | 5.5 |
| Southwest zone | 1090 | 9.3 |
| Lake zone | 2993 | 25.5 |
| Eastern zone | 2238 | 19.1 |
| Central zone | 1164 | 9.9 |
| Zanzibar | 362 | 3.1 |

***Frequency indicates a weighted count**
