# Supplementary material for: Factors associated with underweight, overweight, and obesity in reproductive age Tanzanian women
Source: PLoS One. 2020 Aug 24;15(8):e0237720. doi: 10.1371/journal.pone.0237720 (PMC7444815; doi:10.1371/journal.pone.0237720)
Supplement: S2 Table — (DOCX) [file pone.0237720.s002.docx]

**S2 Table. Factors associated underweight, overweight and obesity among reproductive age women in Tanzania, TDHS 2015–16**

| **Variables** | **Model I**  **(null model)*** | **Model II *** | | | **Model III*** | | | **Model IV*** | | |
| --- | --- | --- | --- | --- | --- | --- | --- | --- | --- | --- |
|  |  | **Underweight** | **Overweight** | **Obesity** | **Underweight** | **Overweight** | **Obesity** | **Underweight** | **Overweight** | **Obesity** |
|  |  | **RRR (95% CI)** | **RRR (95% CI)** | **RRR (95% CI)** | **RRR (95% CI)** | **RRR (95% CI)** | **RRR (95% CI)** | **RRR (95% CI)** | **RRR (95% CI)** | **RRR (95% CI)** |
| **Socioeconomic factors** |  |  |  |  |  |  |  |  |  |  |
| Women’s education |  |  |  |  |  |  |  |  |  |  |
| No schooling |  | 1.00 | 1.00 | 1.00 |  |  |  | 1.00 | 1.00 | 1.00 |
| Primary school |  | 1.18 (0.85, 1.63) | 1.20 (0.92, 1.55) | 1.42 (0.99, 2.02) |  |  |  | 1.22 (0.88, 1.69) | 1.26 (0.97, 1.64) | 1.60 (1.12, 2.28) |
| Secondary and above |  | 1.21 (0.85, 1.72) | 1.44 (1.09, 1.92) | 1.83 (1.25, 2.66) |  |  |  | 1.16 (0.81, 1.67) | 1.48 (1.11, 1.96) | 1.79 (1.23, 2.61) |
| Women’s employment |  |  |  |  |  |  |  |  |  |  |
| No employment |  | 1.00 | 1.00 | 1.00 |  |  |  | 1.00 | 1.00 | 1.00 |
| Formal employment |  | 0.75 (0.54, 1.05) | 1.19 (0.96, 1.49) | 1.37 (1.05, 1.80) |  |  |  | 0.77 (0.55, 1.08) | 1.23 (0.98, 1.53) | 1.50 (1.14, 1.98) |
| Informal employment |  | 0.77 (0.63, 0.94) | 0.99 (0.85, 1.17) | 1.22 (0.99, 1.51) |  |  |  | 0.79 (0.64, 0.96) | 1.00 (0.85, 1.17) | 1.27 (1.03, 1.58) |
| Marital status |  |  |  |  |  |  |  |  |  |  |
| Not married |  | 1.00 | 1.00 | 1.00 |  |  |  | 1.00 | 1.00 | 1.00 |
| Currently married |  | 0.64 (0.47, 0.88) | 1.49 (1.15, 1.93) | 1.81 (1.27, 2.58) |  |  |  | 0.59 (0.43, 0.82) | 1.47 (1.13, 1.91) | 1.78 (1.25, 2.54) |
| Formerly married |  | 0.66 (0.43, 1.01) | 1.21 (0.88, 1.67) | 1.58 (1.05, 2.37) |  |  |  | 0.64 (0.41, 0.98) | 1.19 (0.87, 1.64) | 1.56 (1.03, 2.34) |
| Household wealth status |  |  |  |  |  |  |  |  |  |  |
| Poor |  | 1.00 | 1.00 | 1.00 |  |  |  | 1.00 | 1.00 | 1.00 |
| Middle |  | 0.75 (0.57, 0.99) | 1.56 (1.17, 2.09) | 2.08 (1.25, 3.46) |  |  |  | 0.75 (0.57, 1.00) | 1.51 (1.13, 2.02) | 1.89 (1.14, 3.17) |
| Rich |  | 0.73 (0.57, 0.92) | 2.58 (2.01, 3.31) | 6.36 (4.11, 9.85) |  |  |  | 0.77 (0.60, 1.00) | 2.31 (1.78, 3.03) | 4.77 (3.03, 7.50) |
| **Demographic factors** |  |  |  |  |  |  |  |  |  |  |
| Women’s age |  |  |  |  |  |  |  |  |  |  |
| 15–24 years |  | 1.00 | 1.00 | 1.00 |  |  |  | 1.00 | 1.00 | 1.00 |
| 25–34 years |  | 0.95 (0.71, 1.26) | 2.26 (1.85, 2.77) | 4.27 (3.20, 5.72) |  |  |  | 0.93 (0.69, 1.23) | 2.19 (1.79, 2.68) | 3.92 (2.93, 5.24) |
| 35–49 years |  | 1.39 (0.97, 2.0) | 3.41 (2.67, 4.35) | 11.1 (8.07, 15.4) |  |  |  | 1.36 (0.94, 1.96) | 3.31 (2.59, 4.23) | 9.94 (7.20, 13.73) |
| Parity |  |  |  |  |  |  |  |  |  |  |
| None |  | 1.00 | 1.00 | 1.00 |  |  |  | 1.00 | 1.00 | 1.00 |
| 1–4 children |  | 0.83 (0.61, 1.13) | 1.18 (0.91, 1.53) | 1.19 (0.84, 1.69) |  |  |  | 0.87 (0.63, 1.20) | 1.19 (0.92, 1.55) | 1.23 (0.86, 1.76) |
| 5+ children |  | 0.70 (0.44, 1.10) | 0.99 (0.71, 1.38) | 0.96 (0.63, 1.45) |  |  |  | 0.71 (0.45, 1.13) | 1.02 (0.73 , 1.42) | 0.99 (0.65, 1.52) |
| **Behavioural factors** |  |  |  |  |  |  |  |  |  |  |
| Listening radio |  |  |  |  |  |  |  |  |  |  |
| No |  | 1.00 | 1.00 | 1.00 |  |  |  | 1.00 | 1.00 | 1.00 |
| Yes |  | 0.88 (0.71, 1.09) | 0.99 (0.81, 1.20) | 1.00 (0.77, 1.30) |  |  |  | 0.87 (0.70, 1.08) | 1.00 (0.82, 1.21) | 1.03 (0.79, 1.35) |
| Read magazine |  |  |  |  |  |  |  |  |  |  |
| No |  | 1.00 | 1.00 | 1.00 |  |  |  | 1.00 | 1.00 | 1.00 |
| Yes |  | 0.91 (0.76, 1.10) | 1.14 (0.99, 1.33) | 1.31 (1.08, 1.57) |  |  |  | 0.95 (0.79, 1.15) | 1.15 (0.99, 1.33) | 1.33 (1.10, 1.60) |
| Watch television |  |  |  |  |  |  |  |  |  |  |
| No |  | 1.00 | 1.00 | 1.00 |  |  |  | 1.00 | 1.00 | 1.00 |
| Yes |  | 0.90 (0.73, 1.10) | 1.29 (1.09, 1.53) | 1.82 (1.45, 2.28) |  |  |  | 0.90 (0.73, 1.10) | 1.26 (1.06, 1.50) | 1.70 (1.35, 2.13) |
| Alcohol use |  |  |  |  |  |  |  |  |  |  |
| No |  | 1.00 | 1.00 | 1.00 |  |  |  | 1.00 | 1.00 | 1.00 |
| Yes |  | 0.81 (0.57, 1.13) | 1.14 (0.93, 1.40) | 1.28 (1.01, 1.63) |  |  |  | 0.93 (0.66, 1.31) | 1.17 (0.96, 1.31) | 1.43 (1.12, 1.82) |
| Smoking |  |  |  |  |  |  |  |  |  |  |
| No |  | 1.00 | 1.00 | 1.00 |  |  |  | 1.00 | 1.00 | 1.00 |
| Yes |  | 4.64 (1.57, 13.76) | 0.45 (0.09, 2.14) | 1.16 (0.32, 4.27) |  |  |  | 4.31 (1.46, 12.74) | 0.43 (0.90, 2.10) | 1.07 (0.29, 3.95) |
| Contraceptive use |  |  |  |  |  |  |  |  |  |  |
| No |  | 1.00 | 1.00 | 1.00 |  |  |  | 1.00 | 1.00 | 1.00 |
| Yes |  | 0.66 (0.52, 0.85) | 1.11 (0.95, 1.30) | 1.15 (0.96, 1.38) |  |  |  | 0.70 (0.54, 0.90) | 1.12 (0.96, 1.31) | 1.19 (0.98, 1.44) |
| **Community-level factors** |  |  |  |  |  |  |  |  |  |  |
| Place of residence |  |  |  |  |  |  |  |  |  |  |
| Urban |  |  |  |  | 1.00 | 1.00 | 1.00 | 1.00 | 1.00 | 1.00 |
| Rural |  |  |  |  | 1.15 (0.99, 1.34) | 0.55 (0.49, 0.62) | 0.29 (0.24, 0.35) | 1.22 (0.99, 1.52) | 0.89 (0.76, 1.06) | 0.70 (0.57, 0.86) |
| Region of residence |  |  |  |  |  |  |  |  |  |  |
| Western zone |  |  |  |  | 1.00 | 1.00 | 1.00 | 1.00 | 1.00 | 1.00 |
| Northern zone |  |  |  |  | 1.16 (0.85, 1.57) | 1.56 (1.20, 2.04) | 2.40 (1.56, 3.69) | 0.79 (0.47, 1.31) | 1.08 (0.76, 1.54) | 1.21 (0.81, 1.82) |
| Southern highlands |  |  |  |  | 0.63 (0.45, 0.89) | 1.03 (0.78, 1.36) | 0.87 (0.55, 1.40) | 0.64 (0.37, 1.11) | 0.82 (0.57, 1.18) | 0.51 (0.34, 0.76) |
| Southern zone |  |  |  |  | 0.81 (0.56, 1.16) | 1.41 (1.05, 1.90) | 1.30 (0.79, 2.14) | 1.00 (0.52, 1.94) | 0.95 (0.64, 1.42) | 1.02 (0.57, 1.81) |
| Southwest zone |  |  |  |  | 0.53 (0.38, 0.75) | 1.14 (0.87, 1.50) | 0.99 (0.63, 1.57) | 0.33 (0.17, 0.61) | 1.12 (0.77, 1.62) | 0.72 (0.46, 1.12) |
| Lake zone |  |  |  |  | 0.99 (0.76, 1.28) | 0.81 (0.64, 1.03) | 0.57 (0.38, 0.87) | 0.84 (0.52, 1.36) | 0.77 (0.55, 1.09) | 0.50 (0.33, 0.75) |
| Eastern zone |  |  |  |  | 0.89 (0.64, 1.23) | 1.58 (1.22, 2.06) | 2.14 (1.41, 3.25) | 0.89 (0.53, 1.49) | 1.12 (0.80, 1.56) | 0.91 (0.62, 1.34) |
| Central zone |  |  |  |  | 1.63 (1.22, 2.16) | 1.09 (0.83, 1.45) | 0.82 (0.51, 1.33) | 1.06 (0.64, 1.75) | 0.86 (0.59, 1.25) | 0.61 90.35, 1.05) |
| Zanzibar |  |  |  |  | 1.47 (1.12, 1.92) | 1.87 (1.46, 2.39) | 3.33 (2.22, 5.00) | 1.03 (0.64, 1.66) | 1.14 (0.82, 1.59) | 1.27 (0.89, 1.82) |
|  | **Model I** | **Model II** | | | **Model III** | | | **Model IV** | | |
| **Random effects** |  |  | | |  | | |  | | |
| ICC | 0.24 (0.20, 0.28) | 0.02 (0.01, 0.08) | | | 0.11 (0.08, 0.16) | | | 0.01 (0.001, 0.16) | | |
| PCV | NA | 89.7% | | | -9.0% | | | 10.0% | | |
| MOR | 1.59 (1.52, 1.66) | 1.85 (1.61, 2.08) | | | 1.21 (1.11, 1.28) | | | 1.21 (1.01, 1.55) | | |
| **Model fitness** |  |  | | |  | | |  | | |
| Log likelihood | -12219 | -6539 | | | -11991 | | | -6483 | | |
| AIC | 24450 | 13197 | | | 24048 | | | 13140 | | |
| BIC | 24494 | 13603 | | | 24293 | | | 13528 | | |

***Model I: fitted without explanatory variables (null model); **Model II: fitted with only individual level variables; ***Model III: fitted with only community level variables; ****Model IV: fitted with both individual and community level variables**

**ICC: intra-cluster correlation; PCV: proportion of change in variance; MOR: median odds ratio; AIC: Akaike information criterion; Bayesian information criterion**
