# Supplementary material for: Factors associated with underweight, overweight, and obesity in reproductive age Tanzanian women
Source: PLoS One. 2020 Aug 24;15(8):e0237720. doi: 10.1371/journal.pone.0237720 (PMC7444815; doi:10.1371/journal.pone.0237720)
Supplement: Attachment — Submitted filename: Response to reviewers.docx [file pone.0237720.s004.docx]

**July 25 2020**

**Professor Hajo Zeeb**

**Scientific Editor**

**PLOS ONE**

**Dear Professor Zeeb,**

**RE: Manuscript resubmission – [PONE-D-20-06054R2]** **Factors associated with underweight, overweight, and obesity in reproductive age Tanzanian women**

Thank you for the invitation to revise our subject-titled manuscript and for the very constructive comments. A revised manuscript (clean and version with track changes) reflecting the following point-by-point response have been submitted for your consideration.

**Comment**: L 275 63.1% is not one-third

**Response:**

**Thank you for the observation! Revision done now (line 250).**

**Comment**: L279-280 - the highlighting of high or low prevalences according to very different grouping characteristics appears awkward (mentioning smoking and Southwest Tanzania in one sentence, while these categories may overlap). Rather make separate sentences and compare prevalences according to joint characteristics, e.g. by economic status.

**Response:**

**Revision done (Line 255-267)**

**Comment**: L314 Simply state that compared to women≤ 24 years of age, older women were more likely to be overweight. Do not repeat all numbers from the table in the text, but perhaps chose one to exemplify.

**Response:**

**Revision done (Line 288-292)**

**Comment**: Overall, my suggestion is to more strongly group the findings regarding overweight and obesity, as the associated factors are very similar, and in the discussion you are doing this already in most sections. The first discussion section, however, should also be more condensed with regard to overweight/obesity findings.

**Response:**

**Revision done (Line 320-326)**

**Comment**: L 316 check the value 2.192

**Response:**

**Revision done (Line 288-292)**

**Comment**: L.355 check the wording (reported does not fit here)

**Response:**

**Now revised (Line 326-327)**

**Comment**: L. 448 an "a" was added - why?

**Response:**

**Now revised (Line 419)**

L 474 Sentence starting "In addition" lacks a verb

**Response:**

**Revision done (Line 442-446)**

We thank the Editor and reviewers for the valuable comments and time in reading our manuscript.

We look forward to your final discussion in due course. Please contact me should you require any further information.

Sincerely,

Kedir Yimam Ahmed, MPH

Corresponding author
